# Indigestion Leads to Depression: Avicenna's Point of View

**DOI:** 10.5812/ircmj.15959

**Published:** 2014-02-08

**Authors:** Malihe Motavasselian, Mandana Tavakkoli Kakhki, Mohammad Reza Shams Ardekani

**Affiliations:** 1 Department of Traditional Medicine, Faculty of Traditional Medicine, Tehran University of Medical Sciences, Tehran, IR Iran; 2 Department of Traditional Medicine, Shahid Beheshti University of Medical Sciences, Tehran, IR Iran

**Keywords:** Depression, Dyspepsia, Medicine Traditional

**Dear Editor,**

Depression is a common mental disorder and leading cause of disability worldwide (1). As a definition, depression may be a mood state, a syndrome or a specific disorder that may be secondary to various medical illnesses (2, 3). Avicenna (980-1037 AD), a scholar in traditional medicine, believed that indigestion can lead to depression (4). Indigestion is a common gastrointestinal problem that consists of nausea, vomiting, heartburn, regurgitation and dyspepsia (5). Functional dyspepsia is one of the most common causes of indigestion (6). In the modern medicine the effect of psychological factors on indigestion has been confirmed (2). In the current study we have demonstrated evidences of the effects of indigestion on depression. A recent prospective study in 2012 has stated that people with Functional gastrointestinal disorders (FGIDs) such as functional dyspepsia who had not suffered from anxiety and depression at baseline significantly developed signs and symptoms of anxiety and depression over 12-year follow-up (7). The probable mechanisms for the relationship between depression and dyspepsia have also been studies. Functional brain imaging study has shown neurobiological link between psychological abnormalities and visceral hypersensitivity in FGIDs (8). Another study has approved the role of serotonin and noradrenalin, the two main neurotransmitters involved in the pathophysiology of depression, in indigestion (9). On the other hand, from the Avicenna's point of view the absorbable part of food converts to "chylous" in the stomach that absorbs via the mesenteric vessels to the liver. Then chylous is converted to "chymous" in the liver. Chymous is composed of four humors including "blood", "phlegm", "yellow bile" and "black bile". The body is also composed of these four humors. Humors enter to the blood circulation and supply food and energy for different organs. Abnormal humors cause functional disturbances in all organs including brain. Therefore the development of depression may be explained by the presence of the abnormal humors in the blood circulation. Production of normal humors requires appropriate composition of the gastric chylous that is delivered to the liver. Some etiological factors affect composition of the gastric chylous including: type of food consumed, the quality of gastric digestion, and also the quality of post-feeding conditions. Hence, indigestion can leads to depression with the following mechanism (4, 10)

The preventive methods have a crucial importance in Iranian Traditional Medicine (ITM). To this end, some items have been considered in association with the three mentioned etiological **factors for indigestion as follows**:

**Dietary habits that cause indigestion**

Over eating, low eating, inappropriate time of eating like eating when stomach is full, eating slow-digesting foods before fast-digesting ones and also eating some foods like milk, watermelon, peach and mushroom

**Gastric Disorders that cause indigestion**

Primary gastric disorders like gastric impairments or secondary gastric disorders like ones due to accumulation of excessive gas in stomach or entering of post nasal discharge to it.

**Post-feeding conditions that cause indigestion**

Lack of sleep, excessive movement, intercourse, taking a shower, exposure to hot or cold weather, and also little or too much drinking after meal (4). If the Avicenna’s perspective would be considered alongside with further complementary investigations especially through observational studies, the prevention of indigestion may lead to significance decrease in the prevalence of depression.
